# High-content analysis identified synergistic drug interactions between INK128, an mTOR inhibitor, and HDAC inhibitors in a non-small cell lung cancer cell line

**DOI:** 10.1186/s12885-024-12057-4

**Published:** 2024-03-12

**Authors:** Sijiao Wang, Juliano Oliveira-Silveira, Gang Fang, Jungseog Kang

**Affiliations:** 1 School of Chemistry and Molecular Engineering at East China Normal University, Shanghai, 200062 China; 2 Center of Biotechnology, PPGBCM, Federal University of Rio Grande Do Sul (UFRGS), Porto Alegre, Rio Grande Do Sul 91501970 Brazil; 3 NYU-ECNU Center for Computational Chemistry at NYU Shanghai, Shanghai, 200062 China; 4 Arts and Science, New York University at Shanghai, Shanghai, 200122 China

**Keywords:** Cancer, Drug combination, Synergism, Drug response, High-content analysis, mTOR inhibitor

## Abstract

**Background:**

The development of drug resistance is a major cause of cancer therapy failures. To inhibit drug resistance, multiple drugs are often treated together as a combinatorial therapy. In particular, synergistic drug combinations, which kill cancer cells at a lower concentration, guarantee a better prognosis and fewer side effects in cancer patients. Many studies have sought out synergistic combinations by small-scale function-based targeted growth assays or large-scale nontargeted growth assays, but their discoveries are always challenging due to technical problems such as a large number of possible test combinations.

**Methods:**

To address this issue, we carried out a medium-scale optical drug synergy screening in a non-small cell lung cancer cell line and further investigated individual drug interactions in combination drug responses by high-content image analysis. Optical high-content analysis of cellular responses has recently attracted much interest in the field of drug discovery, functional genomics, and toxicology. Here, we adopted a similar approach to study combinatorial drug responses.

**Results:**

By examining all possible combinations of 12 drug compounds in 6 different drug classes, such as mTOR inhibitors, HDAC inhibitors, HSP90 inhibitors, MT inhibitors, DNA inhibitors, and proteasome inhibitors, we successfully identified synergism between INK128, an mTOR inhibitor, and HDAC inhibitors, which has also been reported elsewhere. Our high-content analysis further showed that HDAC inhibitors, HSP90 inhibitors, and proteasome inhibitors played a dominant role in combinatorial drug responses when they were mixed with MT inhibitors, DNA inhibitors, or mTOR inhibitors, suggesting that recessive drugs could be less prioritized as components of multidrug cocktails.

**Conclusions:**

In conclusion, our optical drug screening platform efficiently identified synergistic drug combinations in a non-small cell lung cancer cell line, and our high-content analysis further revealed how individual drugs in the drug mix interact with each other to generate combinatorial drug response.

**Supplementary Information:**

The online version contains supplementary material available at 10.1186/s12885-024-12057-4.

## Introduction

Cancer is one of the top causes of human death worldwide. In recent decades, many molecular studies have made it possible to significantly extend the 5-year survival rates of various cancers worldwide, but complete remission is rare when cancer metastasizes due to the inherent development of drug resistance [1, 2]. Combinatorial drug treatment has been an effective way to fight cancer drug resistance, as heterogeneous populations of cancers are efficiently killed by multiple cancer drugs [3, 4].

Several quantitative measures based on different drug combination action models, such as Loewe’s additivity, Bliss independence, or highest single agent (HSA), have been used for synergy screening [5, 6]. Loewe’s additivity model determines the expected combination effect as if the same drugs are mixed. The Bliss independence model considers the effect of individual drugs in combination as independent but competitive. The highest single agent model assumes that the strongest single drug effect is the same as the combined drug effect. Since each model has advantages and disadvantages for determining drug synergy, most drug studies choose one method according to their experimental conditions and needs. Recently, a quantitative method comparing the area under the drug inhibition curve has also been suggested for efficient drug synergy screening [7]. However, despite all these efforts, it is still challenging to carry out systematic drug combination screening due to the large number of possible drug combinations for testing.

Systematic synergy screening has been carried out mostly by two methods, large-scale high-throughput screens [8–10] or model-based computational approaches [11–13]. Recently, a phenotypic screen was also suggested as an effective way to screen drug synergy, in which phenotypic profiles of a series of cancer cells to different cancer drug treatments were deduced by high-content analysis of cell images [14] or by sequencing analysis of RNA transcripts [15]. High-content (HC) analysis normally profiles cellular responses to certain drug treatments using specific protein- or cell morphology-based features from microscope images [16–19]. To do this, cells of interest are first recorded by a microscope and segmented by image analysis software (sup Fig. 1A). Various features describing cell or nucleus morphology, reporter gene intensity, and texture pattern are then extracted from the segmented cell images. Population responses to perturbations are represented by the median or KS statistics of each feature, and top hit perturbations can be identified by ranking specific feature scores. In some cases, medians or KS statistics of all features are used to make phenotypic profiles of perturbations for their classification or further characterization by machine learning algorithms. This method has been used to screen drug candidates [20], regulators of biological processes [21], potential toxicity of drug candidates [22], and many others [16–18, 23]. In drug discovery particularly, it identifies mechanisms of action, targets, and even toxicity of drug candidates by comparing their phenotypic signatures with known compounds or genetic perturbation [24–28]. Cell painting is a good example of such approaches [18, 29, 30]. However, drug combination responses have not been systematically studied by the HC analysis.

Here, we carried out a medium-scale optical drug synergy screening and HC analysis using 12 drugs from 6 different drug classes in the A549 cell line, a human non-small cell lung cancer (NSCLC) cell line, providing an effective platform for drug synergy screening. NSCLC is known to be the major lung cancer type with few effective treatment options [31, 32]. In conclusion, our analysis successfully identified synergism between INK128, an mTOR inhibitor, and HDAC inhibitors, which has been reported previously [33–35]. Furthermore, we found that HDAC, HSP90, and proteasome inhibitors played dominant roles in combination drug responses when they were mixed with MT, DNA, or mTOR inhibitors. This information can be utilized for the rational design of multidrug cocktails in the future.

## Results

### The viability of cancer cells was determined from cell images.

To find synergistic drug combinations targeting non-small cell lung cancer cells, we carried out a drug screen with A549 adenocarcinoma cells using a small set of cancer drugs spanning multiple drug classes. In this screen, we utilized the HC image analysis method to examine whether HC analysis could further empower the current synergistic drug screening regimen. Previously, we generated approximately 700 central dogma tag reporter cell lines for HC analysis and examined their discriminative power in recognizing various drug responses [20]. The reporter cell line expressing H2B-CFP, XRCC5-YFP, and mCherry protein was shown to be the best classifier among 93 reporter cell lines and was successfully used for single-compound drug screening. Thus, we used the same reporter cell line for the current drug synergy screen.

**Fig. 1:**
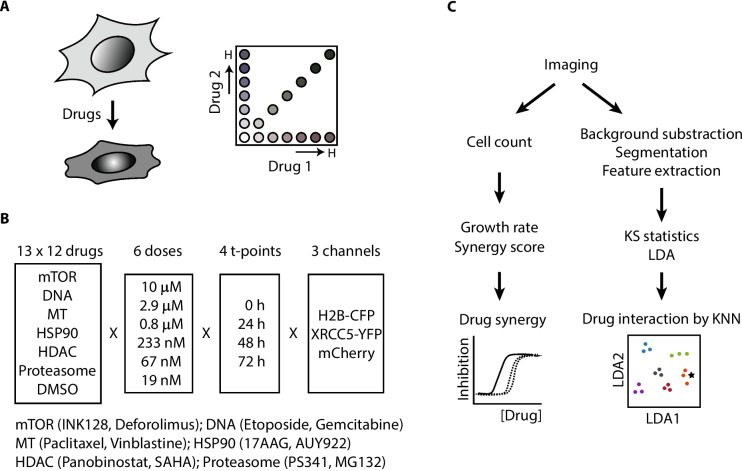
Scheme of drug combinations and their analysis. **A** Each drug was serially diluted by 3.5-fold. Then, two drugs in an equimolar concentration were mixed and treated to cells for combination. **B** 12 drugs, two drugs each in 6 different drug classes, were used for combination. 6 different doses of the equimolar drug mix were treated to cells individually. Then, cells were imaged under microscope by 3 different channels at 4 different timepoints. **C** Cells were directly counted from cell images by Nikon Eclipse software. Then, growth rate and synergy score were calculated from cell numbers for drug synergy screen. Cell images were also used for HS analysis, which included background subtraction, cell segmentation, and feature extraction. Phenotypic profiles were analyzed by KS statistics and LDA for KNN assignment, which allowed us to determine how individual drugs contributed to combination drug responses

Twelve different cancer drug compounds in 6 different categories, including mTOR inhibitors, DNA inhibitors, MT inhibitors, HSP90 inhibitors, HDAC inhibitors, and proteasome inhibitors, were used for combination screening. Because a full factorial combination of serially diluted drugs greatly increased the total number of test combinations, which is a bottleneck of extensive drug synergy screening, we adopted an equimolar drug combination strategy, which would significantly reduce the total number of test combinations. Each drug was prepared from the highest to the lowest concentration by 3.5-fold serial dilution, and two drugs in an equimolar concentration were mixed for combination (Fig. 1A – C). Cells were then treated with a drug mix for 3 days and imaged by a microscope using three different fluorescent channels. Cell numbers were directly counted from microscope images, and cellular drug responses were analyzed by the HC analysis platform, which has been used successfully in our previous study [20]. Optical cell number counting from images was very convenient because no additional reagent treatment was necessary for cell counting and was accurate because cells on images were directly counted (sup Fig. 1B).

**Fig. 2:**
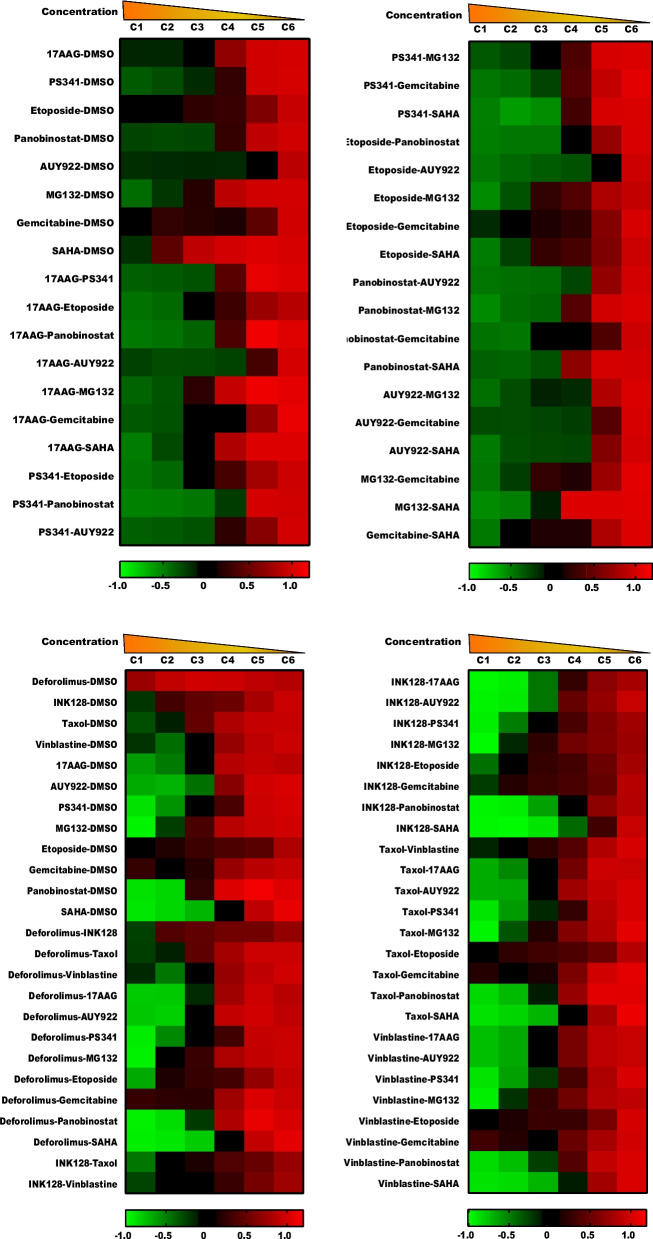
Growth rate after drug combination treatment. Cells were treated with drugs for 3 days and imaged by microscope. Alive cell numbers were extracted from the microscope images and used to calculate growth rate of drug treated cells. Mean values of growth rate were shown as a heatmap (*n* = 4). Cell numbers ranged from 4000 to 10 s. Drug concentration C1 means the highest concentration and C6 means the lowest concentration. Growth rate 1 means no inhibition and -1 means all death

To ensure robust comparison of different cell populations, we further calculated the growth rates of drug-treated cells by comparing their cell numbers with those of DMSO-treated cells [15, 36]. The heatmap of growth rate in Fig. 2 shows that cells with the highest concentration-drug mix mostly died (growth rate, -1), but cells with the lowest concentration-drug mix grew well (growth rate, 1), which validated that our serial dilution method was accurate.

### Drug synergism or antagonism was predicted by synergy scores.

**Fig. 3:**
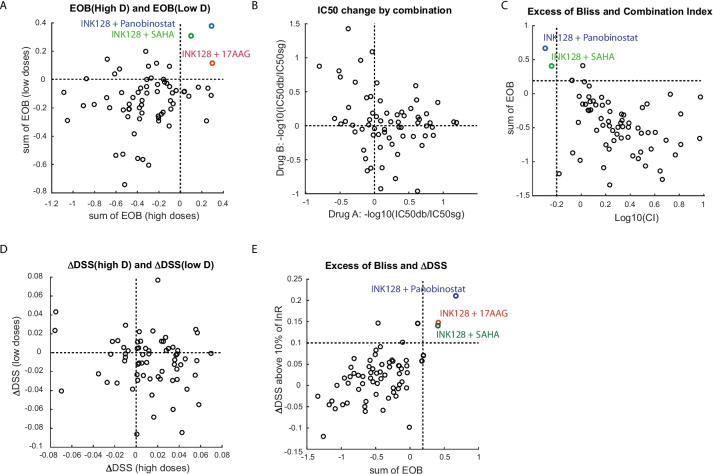
Synergistic combination by EOB, CI, and ΔDSS method. **A** Excess of bliss (EOB) was determined from the growth rate of cells to find synergistic drug combination. The sum of the three low dose EOBs or three high dose EOBs was separately calculated and plotted together. Three combinations in colors exhibited positive EOB in both low and high doses of drug concentration. **B** Change of IC50s by combination. IC50s of two drugs (drug A and B) in combination were examined separately and a fold change (IC50db/IC50sg) of each drug in combination were plotted together. IC50db represents IC50 of one drug in combination treatment and IC50sg represents IC50 of the same drug in single treatment. **C** Sums of all EOBs were plotted together with Loewe’s Combination Index (CI). Two combinations in different colors exhibited EOB, bigger than 0.2, and log10(CI), less than -0.2, suggesting clear synergism. **D** Delta drug sensitivity score (ΔDSS) was determined from the area under the inhibition curve (AUC) of drug treatment to find synergistic drug combination. ΔDSS of the three-low doses or three-high doses were separately calculated and plotted. **E** Sums of all EOBs were plotted together with ΔDSS using regions of more than 10% of full inhibition. Three combinations in different colors exhibited EOB, bigger than 0.2, and ΔDSS, bigger than 0.1, suggesting clear synergism

Combination drug screening often utilizes synergy scores to find drug synergism. We also calculated drug synergy scores according to three different drug action models [5–7]: Bliss independence, Loewe’s additivity, and HSA. For the Bliss independence model, we calculated the excess of Bliss (EOB) for all combinations as a synergy score. EOBs were then summed in three different ways: three higher concentrations, three lower concentrations, or all concentrations. Various drug combinations were shown to be synergistic at three higher or three lower concentrations. Interestingly, two combinations of mTOR/HDAC inhibitors and one combination of mTOR/HSP90 inhibitors were synergistic at all concentrations (Fig. 3A and Table 1). Growth rate plots confirmed that combination treatment increased drug potency (Sup Fig. 2A-C).

**Table 1:** Drug synergy scores via three different methods

mTOR	MT		HSP		PROT		DNA		HDAC			CLASS
*INK*	*PAC*	*VIN*	*AAG*	*AUY*	*PS*	*MG*	*ETO*	*GEM*	*PAN*	*SAHA*	*NAME*	
**CI by Loewe additivity**												
5.33	1.14	1.20	1.06	2.15	1.18	1.49	9.39	**0.95**^c^	**0.84**^**a**^	**0.96**^c^	***DEF***	**mTOR**
	1.66	1.34	1.03	1.47	2.22	3.15	9.25	2.43	**0.51**^**a**^	**0.57**^**a**^	***INK***	
		**0.85**^c^	1.45	2.12	1.16	1.71	1.53	0.98	1.32	1.03	***PAC***	**MT**
			2.18	2.41	1.67	3.52	1.71	0.66	1.25	1.08	***VIN***	
				1.59	1.85	3.06	2.91	3.09	1.96	2.13	***AAG***	**HSP**
					4.59	3.34	1.50	2.64	2.92	2.45	***AUY***	
						1.69	3.72	5.86	2.10	1.16	***PS***	**PROT**
							4.92	6.48	1.81	1.16	***MG***	
								4.40	1.99	3.44	***ETO***	**DNA**
									4.02	6.48	***GEM***	
										1.70	***PAN***	**HDAC**
**Sum of EOB by Bliss independence**												
-0.01	-0.24	-0.24	**0.17**^**b**^	-0.39	-0.12	-0.17	-0.05	-0.42	**0.20**^**a**^	-0.04	***DEF***	**mTOR**
	-0.12	-0.54	**0.41**^**b**^	-0.05	-0.31	-0.20	-0.66	-0.49	**0.67**^**a**^	**0.41**^**a**^	***INK***	
		-0.87	-0.37	-0.65	-0.25	-0.26	-1.09	-0.98	-0.17	-0.15	***PAC***	**MT**
			-0.48	-0.64	-0.41	-0.57	-1.34	-1.18	-0.11	-0.16	***VIN***	
				-0.75	-0.69	-0.86	-0.51	-0.58	-0.59	-0.30	***AAG***	**HSP**
					-1.26	-1.06	-0.46	-0.91	-0.95	-0.43	***AUY***	
						-0.81	-0.81	-0.90	-0.43	0.11^b^	***PS***	**PROT**
							-0.68	-0.75	-0.54	-0.05	***MG***	
								-1.14	-0.49	-0.15	***ETO***	**DNA**
									-0.57	-0.30	***GEM***	
										-0.47	***PAN***	**HDAC**
**ΔDSS by area under curve (%)**												
-9.82	-0.58	-1.61	**5.76**^**b**^	-5.83	0.86	-0.76	-0.57	1.61^c^	**7.07**^**a**^	**3.01**^**c**^	***DEF***	**mTOR**
	3.95	0.87	**14.80**^**b**^	5.91	-2.99	-2.50	-4.96	4.85	**21.09**^**a**^	**14.08**^**a**^	***INK***	
		**2.08**^**c**^	2.45	-6.09	5.71	5.98	-5.47	-4.52	4.68	2.77	***PAC***	**MT**
			2.90	-3.76	4.94	1.72	-2.53	-5.76	10.18	4.64	***VIN***	
				2.41	0.43	-0.31	7.28	5.92	4.44	1.88	***AAG***	**HSP**
					-7.06	-1.59	11.43	0.79	2.58	2.61	***AUY***	
						-3.68	-1.82	0.54	5.03	9.11^b^	***PS***	**PROT**
							2.13	-0.90	3.92	5.67	***MG***	
								-3.66	8.33	2.89	***ETO***	**DNA**
									3.04	-1.14	***GEM***	
										-0.93	***PAN***	**HDAC**

Drug synergy scores were calculated by three different ways, combination index (CI) by Loewe additivity model, sum of excess of bliss (EOB) by Bliss independence model, and delta drug sensitivity score (ΔDSS) by area under curve method. Synergistic interaction gave CI less than 1, EOB more than zero, and ΔDSS more than zero. Drug combinations which showed synergism by all three methods were indicated by “ ^a^ ”. Those which showed synergism by EOB and ΔDSS were indicated by “^b^”. Those which showed synergism by CI and ΔDSS were indicated by “^c^”. 12 drug names: INK128 (INK), paclitaxel (PAC), vinblastine (VIN), 17AAG (AAG), AUY922 (AUY), PS341 (PS), MG132 (MG), etoposide (ETO), gemcitabine (GEM), panobinostat (PAN). Six drug classes: mTOR inhibitor (mTOR), microtubule dynamics inhibitor (MT), HSP90 inhibitor (HSP), proteasome inhibitor (PROT), DNA synthesis inhibitor (DNA), histone deacetylase inhibitor (HDAC)

Next, we calculated the combination index as a synergy score by Loewe’s additivity model [5, 6]. Since the combination index (CI) utilized the IC50 value of each drug, we determined the IC50 of each drug from the growth inhibition curve by a nonlinear regression algorithm in GraphPad software. The IC50 values of most drugs were distributed between the 3rd and 4th serial dilution concentrations (Sup Fig. 2D). We next examined whether combination treatment reduced the IC50s of individual drugs. To do this, we compared the IC50 of drug A in combination with the IC50 of drug A in a single treatment. Similarly, we compared the IC50 of drug B in combination with the IC50 of drug B in a single treatment. Many combinations reduced the IC50s of either drug A or B, but few reduced those of both drugs (Fig. 3B). Next, we calculated Loewe’s CI from the IC50 values of all combinations. Out of 66 combinations, 8 combinations gave CI values less than 1, which would be synergistic (Sup Fig. 4A and Table 1). In Fig. 3C, we compared CIs with summed EOBs. The same two combinations that belonged to the mTOR/HDAC inhibitor combination were synergistic by both methods if we set CI cutoff to 0.6 and EOB cutoff to 0.2.

**Fig. 4:**
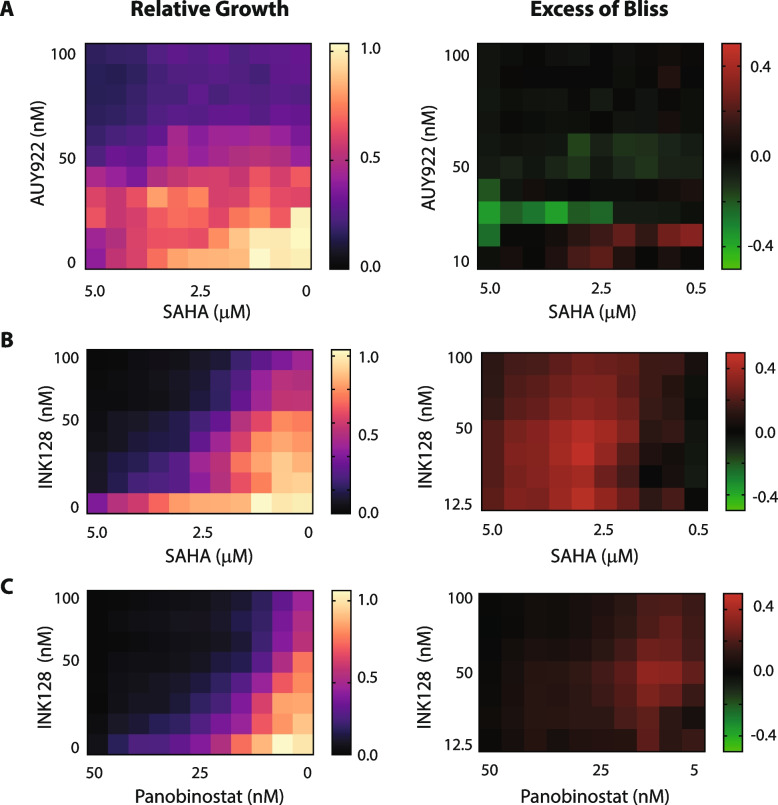
Isobologram of cell viability after drug combination treatment. Cells were treated with (**A**) AUY922 and SAHA, (**B**) INK128 and SAHA, or (**C**) INK128 and panobinostat, for 3 days and cell viability was measured by CCK8 luminescent assay. Relative growth value (left) was determined by comparing their growth with DMSO-treated cells or blank media. Mean values of relative growth were shown as a heatmap (*n* = 4). EOBs (right) were then calculated as previously and their values were also shown as a heatmap. Positive EOBs indicate synergism and negative EOBs indicate antagonism

IC50 is a good indicator of drug potency changes, but it might not detect efficacy changes. One approach that could detect changes in potency and efficacy would be comparing the area under the growth inhibition curve (AUC). A recent study [7] elegantly showed that the drug sensitivity score (DSS), based on normalized AUC by effective inhibition area, accurately predicted drug synergy. Thus, we calculated the DSSs of all combinations and compared them with the DSSs of proper single drugs. Many combinations were shown to be synergistic if we set zero as a cutoff value (Fig. 3D). Interestingly, when we compared **Δ**DSSs with summed EOBs, 3 combinations identified from the previous comparison (Fig. 3A) were also shown to be synergistic if we set EOB cutoff to 0.2 and **Δ**DSS cutoff to 0.1 (Fig. 3E). Consistently, a similar synergistic interaction between mTOR inhibitors and HDAC inhibitors has been reported by other groups [33–35]. To confirm synergism of INK128/SAHA and INK128/panobinostat combination, we have carried out drug matrix-based synergy analysis at 8 × 10 different drug doses. For this, we also included AUY922/SAHA combination as a negative control. As shown in Fig. 4, INK128/SAHA and INK128/panobinostat showed positive EOBs at many different drug doses, but AUY922/SAHA combination didn’t. These data together clearly indicated that mTOR inhibitors were synergistic with HDAC inhibitors, and our HC screening platform using image-based cell counting and equimolar drug concentration mix efficiently identified the known synergistic drug combinations.

### Cellular responses to drug combinations were profiled by HC image analysis.

We next investigated how cancer cells respond to drug combinations. Cellular responses to drug combinations were shown to be the sum of individual drug responses according to protein dynamics studies [37] or shRNA sensitivity signature studies [38], but in-depth phenotypic studies have not been carried out. Thus, we wanted to study how individual drugs in combination would contribute to combination drug responses via HC image analysis. To do this, cells were treated with drug combinations, and images were taken by a fluorescence microscope at the 24-h timepoint and 48-h timepoint for three different reporters: H2B-CFP for nuclear morphology and DNA dynamics, YFP-XRCC5 for XRCC5 protein dynamics, and mCherry for cell morphology. The images were then preprocessed for cellular feature extraction (sup Fig. 1A). A total of 237 features (sup Table 1) representing various information of the three reporters’ intensities, their distribution texture patterns, and cell or nucleus morphologies were extracted from segmented single-cell images, and the population average of each feature was calculated by KS statistics as previously described [20].

We then carried out principal component analysis (PCA) to determine whether different classes of drug compounds generated unique cellular drug responses by their phenotypic profiles. PCA is a mathematical method that can analyze multidimensional data and visualize them in a low-dimensional space [39]. The original coordinates are linearly transformed into new coordinates, in which variations in the data are kept in decreasing order. We applied PCA to all KS feature profile data of drug-treated cells and plotted them in the transformed space using the first two PCA coordinates (sup Fig. 3A&B). The eigenvalues of the first two coordinates indicated that 59% of the total variation was displayed for the first data set and 73% for the second data set. However, we failed to observe distinct cellular responses by drugs of the different classes such that different drug class profiles significantly overlapped.

**Fig. 5:**
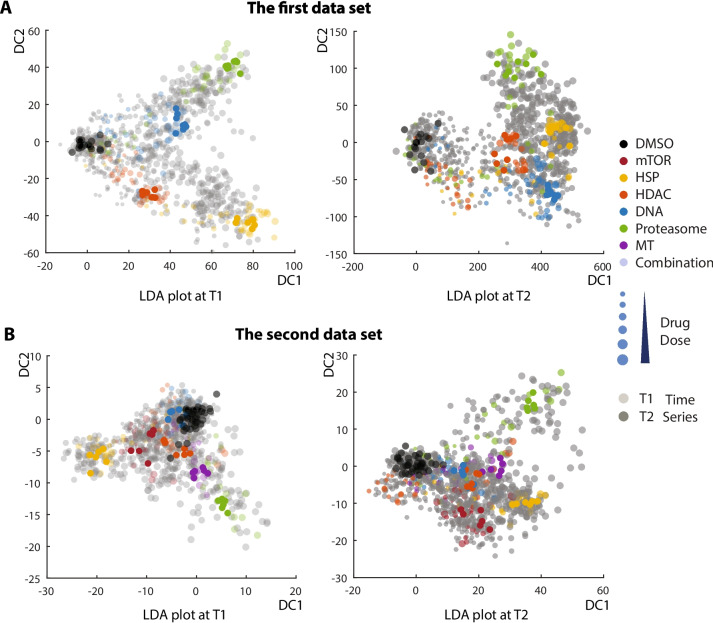
LDA plots using phenotypic profiles of KS statistics after drug combination treatment. The HC analysis extracted cellular features from images of cells treated with different drug compounds at multiple doses. Phenotypic profiles of each drug treatment were calculated by KS statistics and analyzed by LDA. Two independent experiments (A and B) were carried out to test different combinations of drugs. The first data set (A) included all possible combinations of HSP90 inhibitors, HDAC inhibitors, DNA inhibitors, and proteasome inhibitors. The second data set (B) included all possible combination of the previous drugs together with mTOR inhibitor or MT inhibitors. Two different timepoints such as 24-h and 48-h treatment were analyzed separately and their distinct phenotypic profiles were shown by the first two discriminant components. Single treatments were distinguished by different colors and combinations by gray color. Different drug concentrations were also distinguished by different sizes, and different timepoints by different transparencies. The single drug treatments with the second highest drug concentration (C2), which were used as the training data set, were highlighted in solid colors

Thus, we carried out a different analysis, linear discriminant analysis (LDA), to maximize the separation of phenotypic profiles from drugs across different classes. LDA allowed us to identify a linear combination of multiple features to obtain new coordinates, in which drug profiles within the same classes would separate less but drug profiles across the different classes would separate more [40, 41]. The model identified by training objects could be further applied to test new objects for their classification. For the LDA training data set, we used all single drug treatments at high concentrations because those drug treatments generated distinct cellular responses. After finding the clustering model of our training drug set, we applied the same model to all profile data and plotted them in the transformed space using the first two LDA components. In this approach, we clearly observed good clustering of cellular drug responses within the same drug classes but separating across different drug classes (Fig. 5A&B).

### The combination drug response was mostly determined by dominant drug phenotypes.

**Fig. 6:**
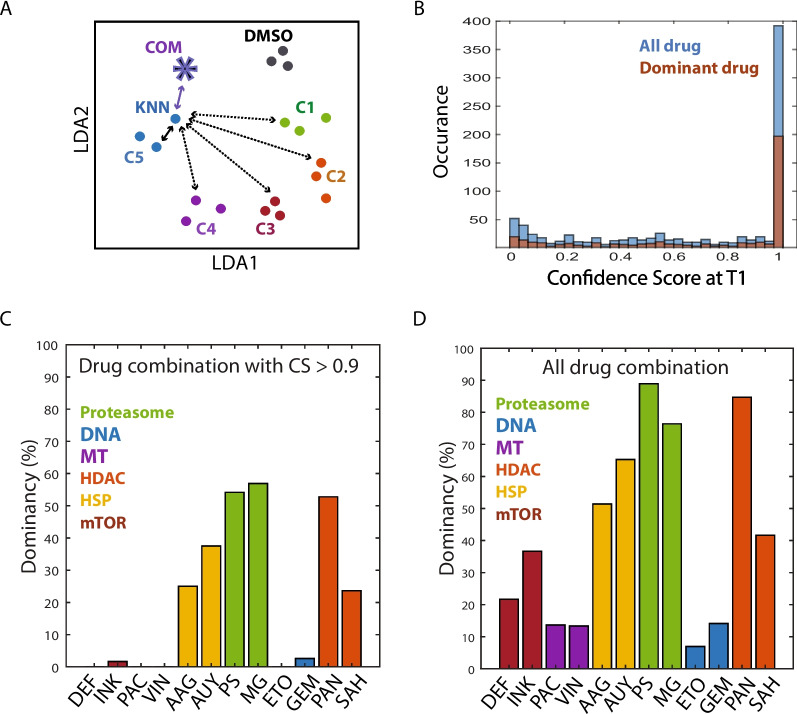
Drug interaction for combination response. **A** Illustration of KNN assignment and confidence score determination. Phenotypic profile of each drug combination was compared with those of the single drug treatment and single drug KNN of each drug combination (star) was assigned by the shortest Euclidean distance. If the drug class of KNN is the same as the drug class of one drug in combination, the drug was considered as dominant. Confidence score was then determined by the Bayes’ theorem as the earlier study [20]. The distance among the same drug classes was compared with the distance between the different drug classes to calculate confidence score. The greater the distance between combination drug and its KNN was, the less confident the combination response was similar to KNN response. **B** Confidence score histogram for 24-h timepoint data set. Out of 872 total assignment, about half gave more than 0.96 confidence score. The combinations showing dominance behavior were shown in brown. **C-D** Statistics of drug dominancy at 24-h timepoint. Percentages of drug combinations showing dominant behavior was shown in a bar graph, using drug combinations with CS higher than 0.9 confidence score (**C**) or all combinations (**D**). 12 drug names: deforolimus (DEF), INK128 (INK), paclitaxel (PAC), vinblastine (VIN), 17AAG (AAG), AUY922 (AUY), PS341 (PS), MG132 (MG), etoposide (ETO), gemcitabine (GEM), panobinostat (PAN), SAHA (SAH)

We next studied how the combination drug response was affected by individual drugs in combination. We envisioned three scenarios. When drug A was dominant and drug B recessive, the A/B combination would follow A drug response, mimicking drug A response profile. When the drug A and drug B were codominant, the A/B combination would exhibit a median response, partially mimicking the single drug response profile of drug A and B. When the drug A and B affected the cell response synergistically, the A/B combination would yield a new and distinct response profile. Thus, by searching single drug k-nearest neighbors (KNNs) of drug combinations and their reliability evaluation by confidence score [20], we could predict how individual drugs contributed to cellular response upon combination drug treatment (Fig. 6A).

We then assigned single drug KNNs for all combinations at the 24-h timepoint (T1) in the LDA transformed space, and their confidence scores were calculated by the training drug distribution distance. Half of the KNN assignments were shown to be reliable since their confidence scores were higher than 90% (Fig. 6B). When we examined the drug combinations with a reliable KNN prediction only (higher than 90% CS), approximately 50% of those drug combinations showed drug dominancy behavior, such that their phenotypic profiles were similar to one dominant drug’s profile. These dominant drugs were mostly proteasome inhibitors, HSP90 inhibitors, and HDAC inhibitors (Fig. 6C). In contrast, DNA inhibitors, mTOR inhibitors, and MT inhibitors failed to maintain their unique drug responses when mixed with other drug compounds. This dominancy pattern was consistently repeated with all drug combination data sets (Fig. 6D). However, we also observed that a significant fraction of drug combinations gave low confidence scores for their KNN assignments, indicating that their profiles were dissimilar to any single drug profile. This might imply that those combination responses could be codominant or potentially synergistic, as we explained earlier. We repeated the analysis with the 48-h timepoint (T2) data set and observed a similar pattern (sup Fig. 4B–D). However, we had to be cautious in interpreting the 48-h timepoint data set since increased cell death might obscure distinct drug responses.

**Fig. 7:**
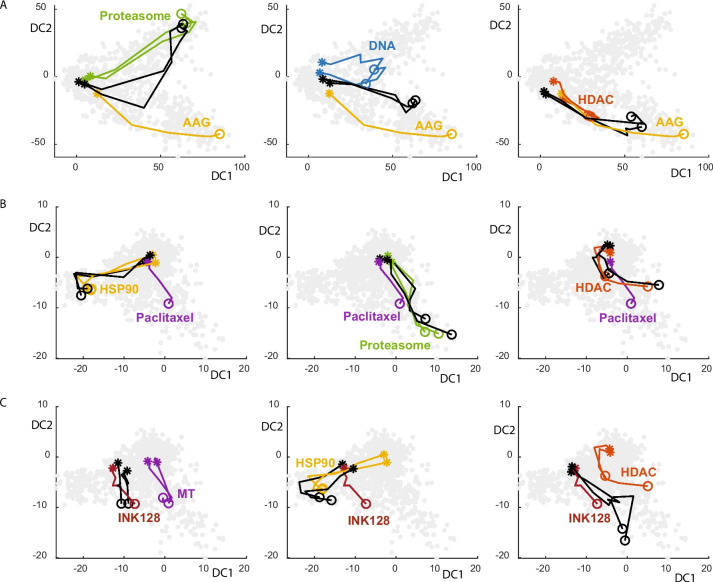
Drug interactions in different concentrations for combination response at 24-h timepoint. **A** Combinations of 17AAG, HSP90 inhibitor, with other drugs. All drug responses were first plotted in gray using two LDA components and mean KS profile (*n* = 4) for all concentrations of the specific drug treatment (C1 through C6) was lined as a concentration trace. Filled asterisks represented the lowest dose drug responses and open circles represented the highest dose drug responses. Single treatment of 17AAG (in yellow), two proteasome inhibitors (in green), two DNA inhibitors (in blue), and two HDAC inhibitors (in light brown), and combination treatment of 17AAG with appropriate inhibitors (in black) were shown separately. **B** Combination of paclitaxel, MT inhibitor, with other drugs. Concentration trace has been made as similarly as (**A**). Single treatment of paclitaxel (in purple), two proteasome inhibitors (in green), two HSP90 inhibitors (in yellow), and two HDAC inhibitors (in light brown), and combination treatment of paclitaxel with appropriate inhibitors (in black) were shown separately. **C** Combination of INK128, mTOR inhibitor, with other drugs. Concentration trace has been made as similarly as (**A**). Single treatment of INK128 (in dark brown), two MT inhibitors (in purple), two HSP90 inhibitors (in yellow), and two HDAC inhibitors (in light brown), and combination treatment of INK128 with appropriate inhibitors (in black) were shown separately

Next, we examined how drug concentrations affected the drug dominancy pattern. To visually examine drug responses by concentration, we aligned the same drug treatment profiles of different concentrations in the LDA-transformed space as a concentration trace (sup Fig. 1A and Fig. 7A-C). The lined single drug treatment is shown in a unique color, but the combination treatment is shown in black. The results again showed three different patterns of drug interactions: dominant-recessive, codominant, and interactive. 17AAG was codominant when it was mixed with DNA inhibitors and HDAC inhibitors (Fig. 7A). The combination drug response lines were positioned in between the two single drug responses, consistent with a median response of two single drug compounds. Interestingly, when it was mixed with proteasome inhibitors, its effect was changed by concentration. At a low concentration, it was codominant, as it was positioned between the two single drug responses, but at a high concentration, its drug response lines overlapped with proteasome inhibitor response lines, which would indicate that 17AAG was recessive to proteasome inhibitors. In the case of paclitaxel (Fig. 7B), it was recessive to HSP90 inhibitors, proteasome inhibitors, and HDAC inhibitors. In the case of INK128 (Fig. 7C), its effect was changed by drug class. It was dominant to MT inhibitors but recessive to HSP90 inhibitors. However, when it was mixed with HDAC inhibitors, which was shown to be a synergistic interaction, the combination phenotype was drastically different from either single drug, especially at the highest concentration, implicating a distinct combination phenotype. It would be interesting to study in the future what molecular changes were responsible for this distinct phenotype.

The drug combination was reported to treat cancer patients more effectively by independent actions, as two effective cancer drugs cured heterogeneous patients better [42]. Thus, multidrug cocktails in which individual drugs elicit strong responses are commonly used in cancer therapeutics, but unexpected drug interactions often hinder their effective treatment. Here, we showed how individual drugs in combination contribute to cellular drug responses by HC analysis and suggested the priority of drug compounds in making multidrug cocktails such as dominant types in lower concentrations, which may reduce cell toxicity. To test our prediction, we prepared three-drug combos with different drug classes and examined cell viability after their treatment. To have similar potency of each drug, we calculated EC20s and EC50s of all drugs from single drug growth curve (Sup Fig. 5A-G) and mixed them at EC20 or EC50 of each drug. The most dominant drug type such as proteasome inhibitor was again shown to be more potent than other inhibitors (Sup Fig. 6), confirming our earlier finding. Thus, our study opens a new possibility that high content analysis of drug interactions can help the rational designing of multidrug cocktails to combat not only drug resistance but also drug toxicity.

## Discussion

A common approach to cancer therapy is monotherapy or combination therapy. Combination therapy is an effective treatment option because it reduces drug resistance development and relieves drug side effects. However, it is not easy to find synergistic drug combinations by high-throughput screening due to a large testing sample size. Here, we developed an image-based high-throughput synergy screening and carried out a pilot screening using 12 chemotherapeutic and targeted cancer drug compounds over 6 different drug classes. The screening identified that mTOR inhibitors showed synergism with HDAC inhibitors. This finding was consistent with several earlier studies. One study showed that this combination greatly increased the death of B-cell acute lymphoblastic leukemia cells compared to single treatment [35]. Another study showed that more than 60 cancer cell lines exhibited synergistic sensitivity to this combination and that breast cancer patient-derived xenograft and BCL-XL plasmacytoma mouse models both exhibited enhanced responses [34]. They further showed that the synergy resulted from enhanced MYC degradation. Another study reported that HDAC/mTOR inhibitors synergized with a HER2 inhibitor to kill pancreatic ductal adenocarcinoma [33]. Furthermore, we also observed a synergistic interaction between mTOR inhibitors and HSP90 inhibitors by the Bliss independence and AUC methods, albeit weak. Consistently, Millson et al. also reported that HSP90 inhibitor treatment often activates HSF1 to develop resistance, but mTOR inhibitor cotreatment sensitizes cells [43]. Another study showed that HSP90 inhibitors were synergistic with dual PI3K/mTOR inhibition in Burkitt lymphoma driven by MYC dysregulation [44]. Last, cisplatin-resistant human bladder cancer cells were also shown to be more sensitized with combined treatment of HSP90 and a PI3K/mTOR dual inhibitor, which was mediated by increased G1 arrest and apoptosis [45]. Therefore, our study clearly proved the effectivity of our platform on a medium-scale drug synergy screening and its potential for a large-scale synergy screening in future.

The synergistic killing of cancer cells by drug combinations has been intensively studied, but individual drug interactions in drug combinations have not been systematically investigated. Here, we studied combination drug responses by using the HC drug profiling method. Our analysis showed that distinct cellular responses were observed by different drug classes, and combination responses could be determined largely by one dominant drug. In our studies, HDAC, HSP90, or proteasome inhibitors played dominant roles in combination drug responses when they were mixed with DNA, MT, or mTOR inhibitors. For instance, proteasome inhibitors such as PS341 were clearly dominant over all other drug compounds at high concentrations (data not shown), but MT inhibitors such as paclitaxel were the opposite (Fig. 7B). We thought that the cell cycle effect played an important role in this behavior. DNA or MT inhibitors normally arrest cells at specific cell cycle stages, which eventually leads to cell death. However, HDAC, HSP90, or proteasome inhibitors directly induce apoptotic cell death regardless of the cell cycle stage. Interestingly, we found that synergistic combinations generated distinct combination responses. When INK128 was mixed with HDAC inhibitors, which was a synergistic interaction according to the cell proliferation assay (Table 1), the combination response followed INK128’s response at a low dose but HDAC’s response at higher doses (Fig. 7C). Since we mixed drug in equal concentrations, it is quite possible that INK128 is more potent at lower concentrations but HDAC is potent and dominant at higher concentration. However, at the maximum dose, the combination response was drastically different from both the INK128 and HDAC inhibitor responses. Interestingly, the drug concentrations that gave the highest synergy scores were low doses (Fig. 4). Thus, INK128 and HDAC inhibitors exhibited interesting concentration-dependent drug interactions, which need further study for their interesting molecular behavior and drug potency.

We showed in this study that HC analysis could enhance the effectiveness of current synergistic drug screening regimens by providing additional information on drug interactions in combination. Our HC image analysis used various cellular features, such as cell or nucleus morphology, DNA dynamics, XRCC5 protein intensity, and its distribution pattern (sub Table 1). How each feature contributes to the cellular drug response is an important question that we would like to answer in the future. In the current study, however, we have not been able to address this issue since we used the LDA classification method, which transformed individual features to maximize distance over different types of drugs but minimize distance among the same types of drugs. It was clear that XRCC5 protein dynamics played an important role in discriminating different drug responses since the XRCC5 reporter cell line was the top ranked cell line for its discriminative power, but we did not have any evidence that its power could result from XRCC5’s DNA damage repair role. Therefore, it would be an important topic to study in the future.

## Conclusion

Combinatorial drug treatment has been an effective way to fight cancer drug resistance. But there are few studies that systematically screen multidrug combos to treat cancers. Here, we have set up an optical screening platform to screen drug synergism and identified a synergistic interaction between mTOR inhibitors and HDAC inhibitors. Furthermore, we have discovered interesting drug interaction patterns between individual drugs in pairs such as dominant, codominant, recessive, or synergistic. Since unexpected drug interactions often hinder their effectiveness targeting cancer cells, drug interaction studies by our optical screening and analysis could provide an important insight in the field of therapeutic drug combination studies.

## Methods

### Cell culture and drug treatment

The A549 adenocarcinoma cell line was purchased from ATCC and genetically engineered to express H2B-CFP, YFP-XRCC5, and mCherry as described previously [20]. Cells were cultured in Dulbecco's modified Eagle's medium (DMEM, Invitrogen Gibco) supplemented with 10% fetal bovine serum (FBS, SORFA), 100 U/mL penicillin, and 100 μg/mL streptomycin (MesGen Biotech) in a 37°C, 5% CO2 incubator. All cells were tested for the absence of mycoplasma. For drug combination screening, we seeded 3000 cells in each well of a 384-well optical plate (Thermo Fisher Scientific, plastic) by brief centrifugation and sealed them with Breathe-Easy® sealing membrane (Sigma–Aldrich). After cells adhered to the plates, we took images of the cells by a Nikon Eclipse Ti-E microscope with a 10X objective lens every 24 h for 3 days.

Single drugs were prepared in six different concentrations by 3.5-fold dilution, and two different single drugs in equimolar concentrations were combined as a drug mix. The drug mix was then treated to replicates of cells by a MINI 96 (INTEGRA) multichannel pipette (1st data set of 8 × 8 drugs, 17AAG, AUY922, PS341, MG132, etoposide, gemcitabine, panobinostat, SAHA, has 4 duplicates; 2nd data set of 4 × 12 drugs, deforolimus, INK128, paclitaxel, vinblastine, 17AAG, AUY922, PS341, MG132, etoposide, gemcitabine, panobinostat, SAHA, has 3 duplicates). After 3 days of drug treatment, cells were fixed with 75% ethanol, stained with Hoechst 33342 (MESGEN), and counted by the bright spot detection function of the Nikon Eclipse HC software. Initial cell counting was carried out similarly but using H2B-CFP signal in live cell images. When cell numbers in one of four (or three) biological replicates significantly diverged from their mean by more than one standard deviation, we removed it from the following analysis as outlier.

For luminescent-based cell viability assay, cells were seeded in a 96-well plate by brief centrifugation and appropriate drugs were treated by multichannel pipette (Sup Table 2). After three days of drug treatment, CCK8 (cell counting kit-8, GLPBIO) assay was carried out and luminescent light was recorded by a microplate reader (Bio-Rad).

### GR, CI, EOB, DSS, and RG calculation

We calculated the growth rate of drug-treated cells based on an earlier report [15, 36], which allows robust comparison of cell survival among drug-treated samples. The normalized growth rate formula is:

$$GR={^{2}({log}_2(X_c/X_o)/{log}_2(X_{dc}/X_o)))-1}$$

where X_c_ is the cell count after 72 h of drug treatment, X_o_ is the cell count before drug treatment, and X_dc_ is the cell count after 72 h of no drug treatment.

The three methods described below were used to measure the synergy of the two drugs. The combination index (CI) according to the Loewe additivity model was calculated by the following formula [46]:

$$CI = {IC50}_{a(a+b)}/{IC50}_{a} + {IC50}_{b(a+b)}/{IC50}_{b}$$

where IC50 is the half maximal inhibitory concentration of a given drug, IC50_a(a + b)_ is the IC50 of drug A in the combination of Drug A and Drug B, IC50_b(a + b)_ is the IC50 of drug B in the combination of drug A and drug B, and IC50_a_ and IC50_b_ are the IC50 of drug A and drug B, respectively. CI < 1 is synergism; CI = 1 is an additive effect; CI > 1 is antagonism. Drug IC50s were calculated by the nonlinear regression algorithm of GraphPad software using growth rates in given log drug concentrations.

The excess of Bliss independence (EOB) was calculated by the following formula:

$${EOB}_{GR} =(1-{GR}_{com})-(1-{GR}_{a})-(1-{GR}_{b})+(1-{GR}_{a})\times (1-{GR}_{b})$$

where GR_com_ is the growth rate of the drug combination, and GR_a_ and GR_b_ are the growth rates of drug A and drug B, respectively. EOB > 0 is synergism; EOB < 0 is antagonism.

The drug sensitivity score (DSS) was calculated as described in a previous report [7]. In short, the dose–response function y as a continuous function of the dose x was modeled using a nonlinear regression function as follows:

$$y = d+\frac{a-d}{1+{10}^{b(c-x)}}$$

where a is the maximal response, b is the slope of the curve, c is the IC50, and d is the minimal response. The area under the curve (AUC) was then calculated by the following formula over the selected concentration range from x_1_ to x_2_:

$$AUC = {\int }_{x1}^{x2}y(x)dx = Y({x}_{2}) - Y({x}_{1})$$

where the integral function of the dose–response can be expressed as

$$Y(x) = \frac{(a-d){log}_{10}(1+{10}^{b(c-x)})}{b} + ax$$

The DSS was finally calculated after normalization of the AUC as follows:

$$DSS = \frac{AUC - t\times ({x}_{2}-{x}_{1})}{(100-t)\times ({C}_{max}-{C}_{min})}$$

where t is the minimum activity level at which integration begins (10% by default), and C_max_ and C_min_ are the maximum and minimum drug concentrations, respectively, in which the drug was screened.

$$\boldsymbol{\triangle} DSS=DSS_{combination}-max\ [DSS_{drug\ A},\ DSS_{drug\ B}]$$

**Δ**DSS > 0 indicates synergism.

Relative growth (RG) value for CCK8 growth assay is calculated by the next formula:

$$RG= ({LS}_{d}- {LS}_{m})/({LS}_{c}-{LS}_{m})$$

where LC_d_ is the luminescence signal value of 72 h-drug treated cells, LC_m_ is the luminescence signal value of blank cell culture media, and LC_c_ is the luminescence signal value of 72 h-DMSO treated cells.

### HC analysis

Image background subtraction, segmentation, feature extraction, and phenotypic profiling by KS statistics were carried out as described previously [20]. The following analyses of KS profiles of drug-treated cells, such as principal components analysis, linear discriminant analysis, k-nearest neighbor classification, and confidence score calculation, were also carried out as described previously [20]. All MATLAB codes and data can be found in the supplemental data.

## Supplementary Information

**Additional file 1: Sup Figure 1.** Overall scheme of HC image analysis. (A) HC analysis of drug combination. Cells with or without drugs were first imaged by microscope. Background subtraction, cell segmentation, and feature extractions were carried out as similarly as our earlier studies [20] to give phenotypic feature profiles. Population average of each feature was calculated by KS statistics and concatenated as KS profile. After LDA, each concentration of drug treatment (C1 through C6) was lined as a concentration trace. (B) Cell counting by Nikon Element HC analysis software. DNA was stained, imaged, and counted by bright spot detection function of Nikon element. Representative images were shown. Error bar, 10μm.

**Additional file 2: Sup Figure 2.** Synergistic combination. Growth rate curves of (A) INK128/SAHA, (B) INK128/panobinostat, and (C) INK128/17AAG treatments. Means of growth rates were shown with standard deviation (n=4). All three combinations were shown to be synergistic by more than one synergy model. Six different drug concentrations from the highest (C1) to the lowest (C6) were made by 3.5-fold serial dilution. Highest concentration is 10 µM for INK128, 200 µM for SAHA, and 5 µM for panobinostat. (D) Violin plot of drug IC50s. Drug IC50s for single or double treatments were determined from the growth rate inhibition curve using GraphPad software.

**Additional file 3: Sup Figure 3.** PCA plot using phenotypic profiles of KS statistics after drug combination treatment. The HC analysis extracted cellular features from images of cells treated with different classes of drugs in multiple concentrations. Phenotypic profiles of each drug treatment were calculated by KS statistics and analyzed by PCA. Two independent experiments testing different sets of drug combinations, as described in Figure 4, were analyzed separately and their distinct phenotypic profiles were shown in the two-dimensional space by the first two principle components (A and B). Single treatments were distinguished in different colors and combination in gray color. Different drug concentrations were also distinguished by different sizes, and different timepoints by different transparencies. DMSO control were diluted from the highest concentration, 0.1%, as similarly as other drugs.

**Additional file 4: Sup Figure 4.** (A) Venn diagram summary of drug synergy screen. Drug synergy was determined by three different methods, Loewe additivity, Bliss independence, and area under curve. Among 66 combinations, 8 combinations were synergistic by Loewe additivity, 6 by Bliss independence, and 41 by AUC. 3 combinations are synergistic by all three methods. (B) Histogram of confidence score. Confidence scores of KNN assignment for all combinations at 48-hr timepoint were shown in a histogram. KNN assignments with dominant behavior were shown in brown. (C-D) Drug dominancy statistics of 48-hr timepoint data set. Percentages of drug combinations showing dominant behavior was shown in a bar graph, using drug combinations with CS higher than 0.9 confidence score (C) or all combinations (D). 12 drug names: INK128 (INK), paclitaxel (PAC), vinblastine (VIN), 17AAG (AAG), AUY922 (AUY), PS341 (PS), MG132 (MG), etoposide (ETO), gemcitabine (GEM), panobinostat (PAN).

**Additional file 5 Sup Figure 5.** Relative growth curves of single drug treatment. Cells were treated with (A) AUY922, (B) PS341, (C) gemcitabine, (D) paclitaxel, (E) INK128, (F) SAHA, or (G) panobinostat, for 3 days and cell viability was measured by CCK8 luminescent assay. Relative growth value was determined by comparing their growth with DMSO-treated cells or blank media. Mean values of relative growth were plotted with standard deviation (n=4). EC50 values were calculated by GraphPad prism.

**Additional file 6: Sup Figure 6.** Relative growths after drug combination treatmentd and their EOBs. EC20 or EC50 values of each single drug were first deduced from growth rate curve (Sup Figure 5) by GraphPad prism and adjusted values after verification were used. EC20 and EC50: INK128 (17 nM & 96 nM), SAHA (1.5 µM & 5 µM), AUY922 (31 nM & 68 nM), PS341 (10 nM & 15 nM), Gemcitabine (12 nM & 37 nM), and Paclitaxel (50 nM & 500 nM). Drug mixture (single, double or triple) using EC20 level of each drug was then treated to cells for 3 days and cell viability was measured by CCK8 luminescent assay. Relative growth value was determined by comparing their growth with DMSO-treated cells or blank media and EOB was accordingly calculated. Mean values of relative growth (n=4) and EOBs were shown as a heatmap.

**Additional file 7: Sup Table 1.** List of all features in high-content analysis. 239 features representing morphology, texture, and intensity information are described.

**Additional file 8: Sup Table 2.** Relative growth rates of survival assays. All combinations of survival assay using CCK8 reagents were described.

## Data Availability

All data generated or analyzed during this study are included in this published article and its supplementary files.

## Abbreviations

HC: High-content
NSCLC: Non-small cell lung cancer
MT: Microtubule
DNA: Deoxynucleic acid
HSP90: Heat shock protein 90
HDAC: Histone deacetylase
mTOR: Mammalian target of rapamycin
HSA: Highest single agent
EOB: Excess of bliss
CI: Combination index
AUC: Area under curve
DSS: Drug sensitivity score
KS: Kolmogorov-Smirnov
GR: Growth rate
PCA: Principle component analysis
LDA: Linear discriminant analysis
KNN: K-nearest neighbor
XRCC5: X-ray repair cross complementing 5
CFP: Cyan fluorescent protein
YFP: Yellow fluorescent protein
