# Supplementary material for: High-content analysis identified synergistic drug interactions between INK128, an mTOR inhibitor, and HDAC inhibitors in a non-small cell lung cancer cell line
Source: BMC Cancer. 2024 Mar 12;24:335. doi: 10.1186/s12885-024-12057-4 (PMC11542337; doi:10.1186/s12885-024-12057-4)
Supplement: Supplementary file 1 — Additional file 1: Sup Figure 1. Overall scheme of HC image analysis. (A) HC analysis of drug combination. Cells with or without drugs were first imaged by microscope. Background subtraction, cell segmentation, and feature extractions were carried out as similarly as our earlier studies [20] to give phenotypic feature profiles. Population average of each feature was calculated by KS statistics and concatenated as KS profile. After LDA, each concentration of drug treatment (C1 through C6) was lined as a concentration trace. (B) Cell counting by Nikon Element HC analysis software. DNA was stained, imaged, and counted by bright spot detection function of Nikon element. Representative images were shown. Error bar, 10μm. [file 12885_2024_12057_MOESM1_ESM.pdf]

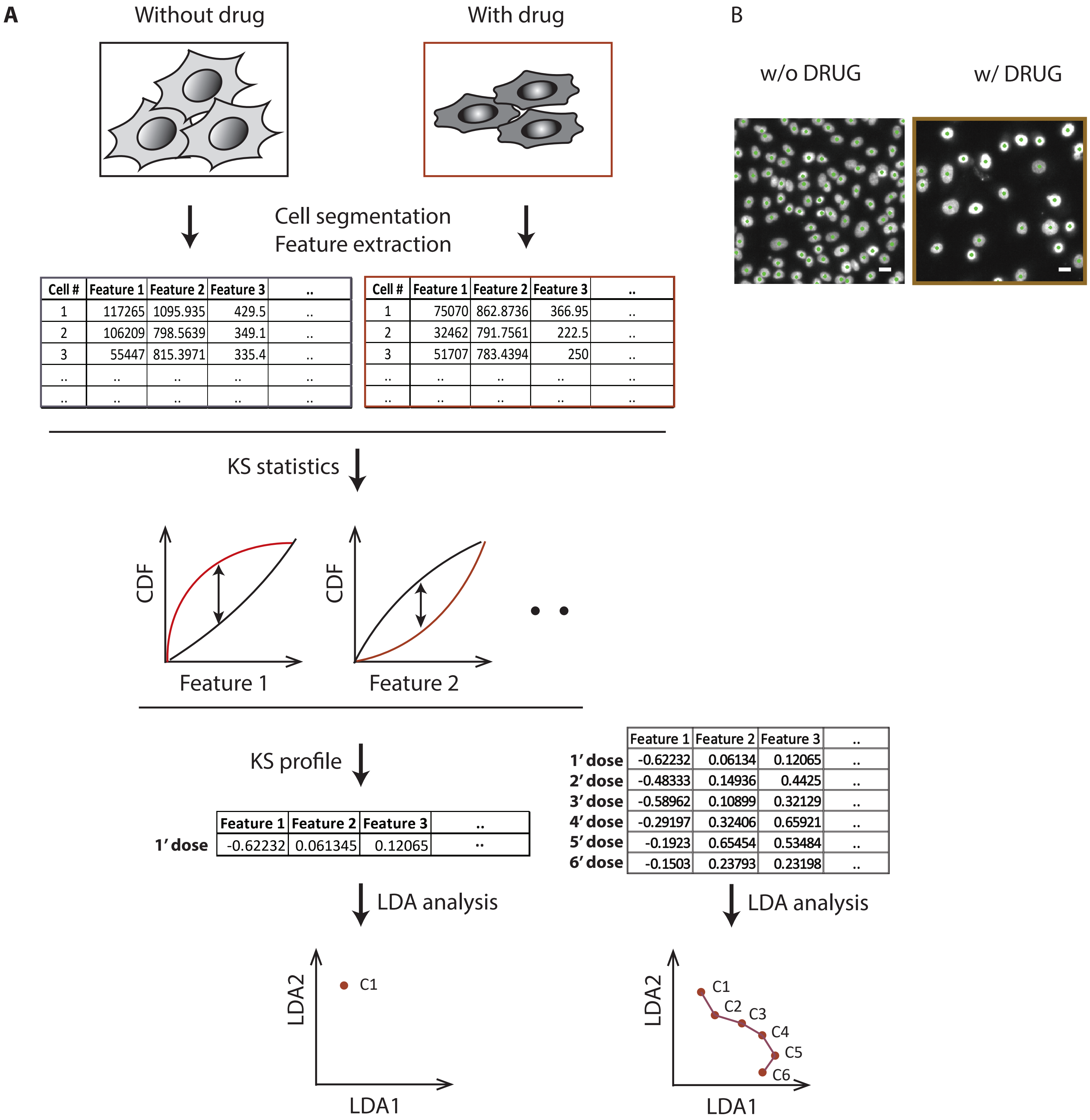
A
Without drug
With drug
B
w/o DRUG
w/ DRUG
Cell segmentation
Feature extraction
Cell # | Feature 1 | Feature 2 | Feature 3 | ..
1 | 117265 | 1095.935 | 429.5 | ..
2 | 106209 | 798.5639 | 349.1 | ..
3 | 55447 | 815.3971 | 335.4 | ..
Cell # | Feature 1 | Feature 2 | Feature 3 | ..
1 | 75070 | 862.8736 | 366.95 | ..
2 | 32462 | 791.7561 | 222.5 | ..
3 | 51707 | 783.4394 | 250 | ..
KS statistics
CDF
Feature 1
CDF
Feature 2
KS profile
Feature 1 | Feature 2 | Feature 3 | ..
1′ dose | -0.62232 | 0.061345 | 0.12065 | ..
Feature 1 | Feature 2 | Feature 3 | ..
1′ dose | -0.62232 | 0.06134 | 0.12065 | ..
2′ dose | -0.48333 | 0.14936 | 0.4425 | ..
3′ dose | -0.58962 | 0.10899 | 0.32129 | ..
4′ dose | -0.29197 | 0.32406 | 0.65921 | ..
5′ dose | -0.1923 | 0.65454 | 0.53484 | ..
6′ dose | -0.1503 | 0.23793 | 0.23198 | ..
LDA analysis
LDA analysis
LDA2
C1
LDA1
LDA2
C1
C2
C3
C4
C5
C6
LDA1
