# Supplementary figures and images for: High-content analysis identified synergistic drug interactions between INK128, an mTOR inhibitor, and HDAC inhibitors in a non-small cell lung cancer cell line
Source: BMC Cancer. 2024 Mar 12;24:335. doi: 10.1186/s12885-024-12057-4 (PMC11542337; doi:10.1186/s12885-024-12057-4)

A

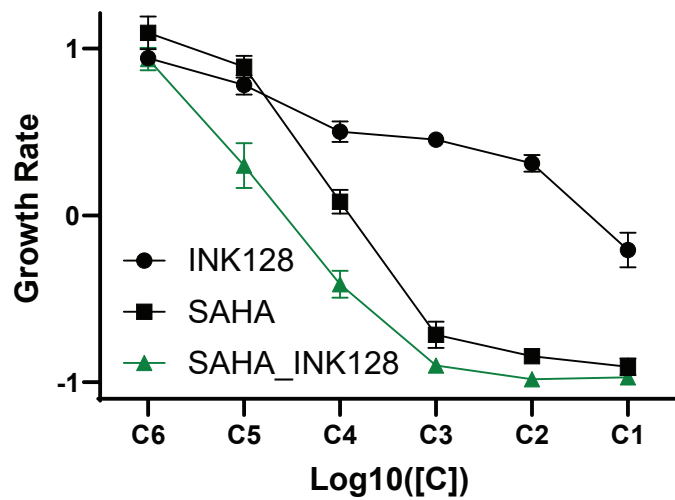

B

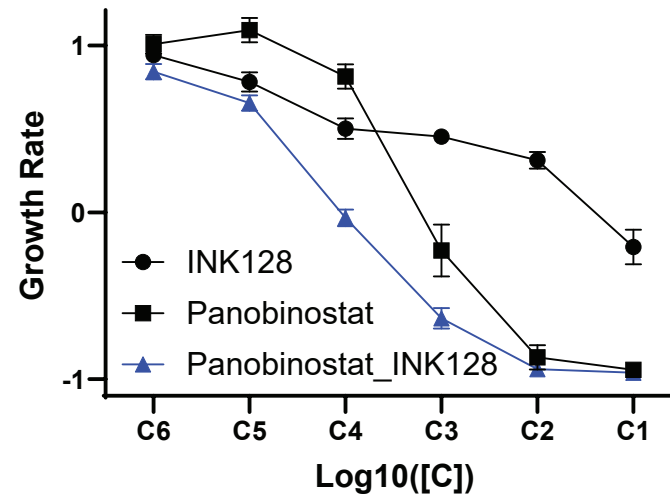

C

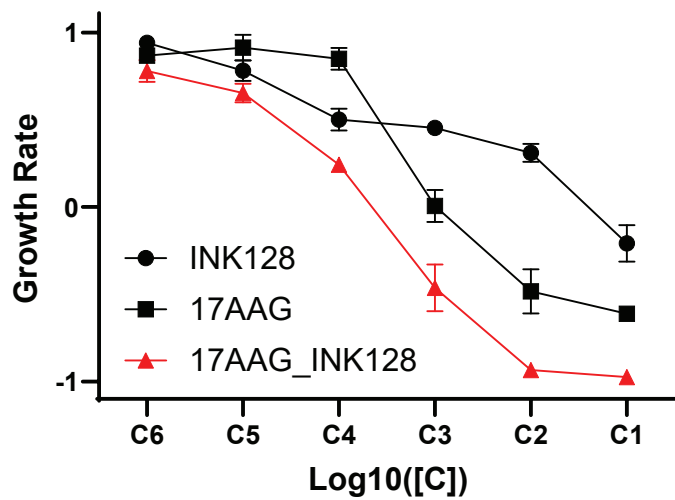

D

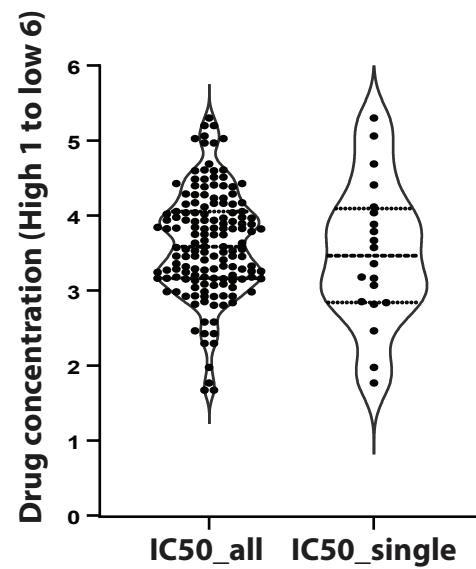

Supplement: Supplementary file 2 — Additional file 2: Sup Figure 2. Synergistic combination. Growth rate curves of (A) INK128/SAHA, (B) INK128/panobinostat, and (C) INK128/17AAG treatments. Means of growth rates were shown with standard deviation (n=4). All three combinations were shown to be synergistic by more than one synergy model. Six different drug concentrations from the highest (C1) to the lowest (C6) were made by 3.5-fold serial dilution. Highest concentration is 10 µM for INK128, 200 µM for SAHA, and 5 µM for panobinostat. (D) Violin plot of drug IC50s. Drug IC50s for single or double treatments were determined from the growth rate inhibition curve using GraphPad software. [file 12885_2024_12057_MOESM2_ESM.pdf]

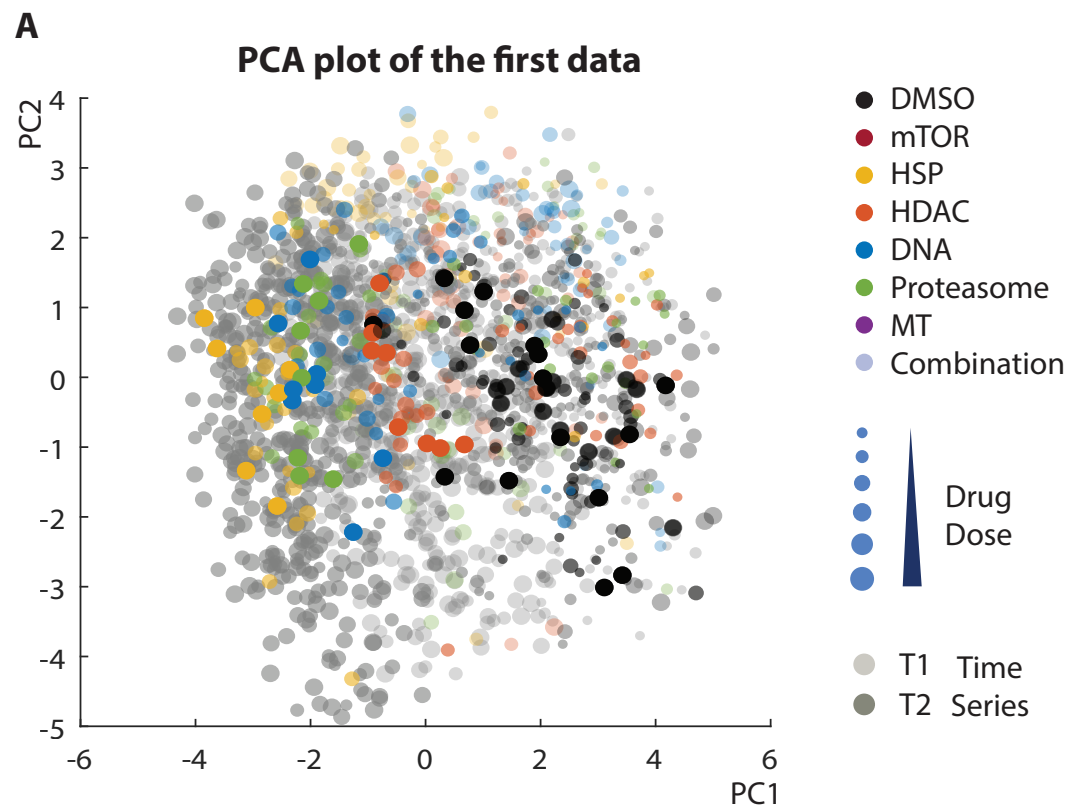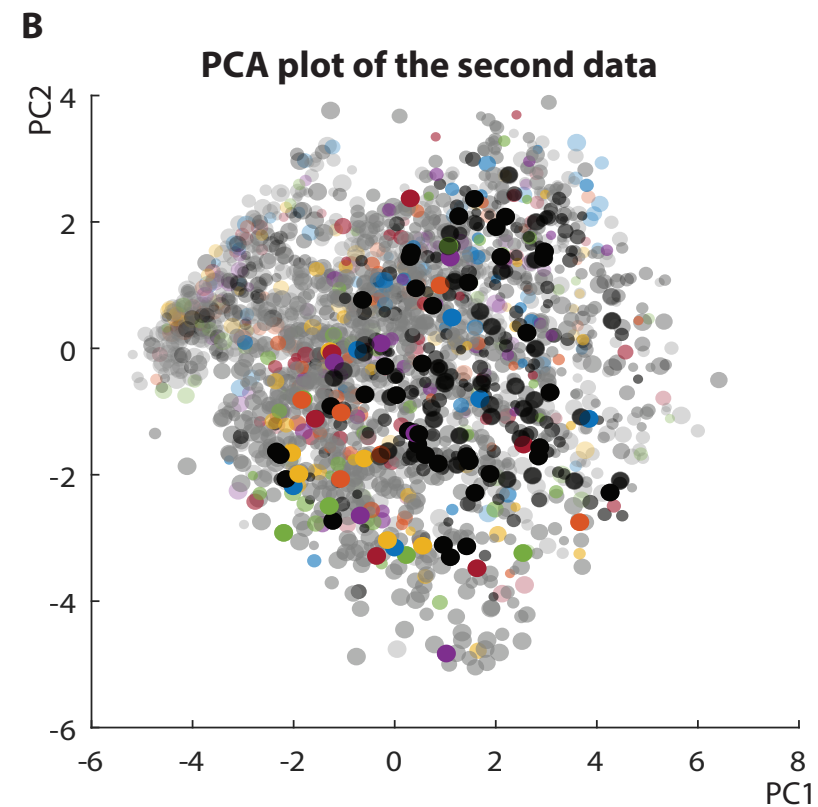

Supplement: Supplementary file 3 — Additional file 3: Sup Figure 3. PCA plot using phenotypic profiles of KS statistics after drug combination treatment. The HC analysis extracted cellular features from images of cells treated with different classes of drugs in multiple concentrations. Phenotypic profiles of each drug treatment were calculated by KS statistics and analyzed by PCA. Two independent experiments testing different sets of drug combinations, as described in Figure 4, were analyzed separately and their distinct phenotypic profiles were shown in the two-dimensional space by the first two principle components (A and B). Single treatments were distinguished in different colors and combination in gray color. Different drug concentrations were also distinguished by different sizes, and different timepoints by different transparencies. DMSO control were diluted from the highest concentration, 0.1%, as similarly as other drugs. [file 12885_2024_12057_MOESM3_ESM.pdf]

A

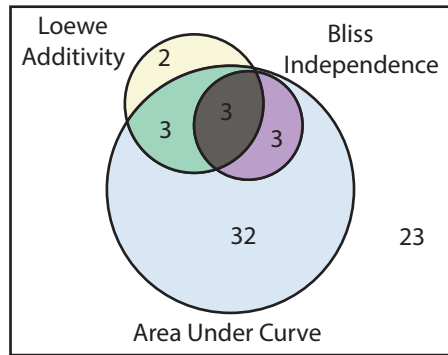

B

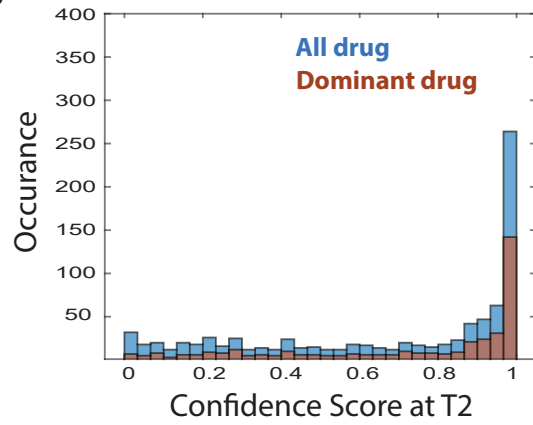

C

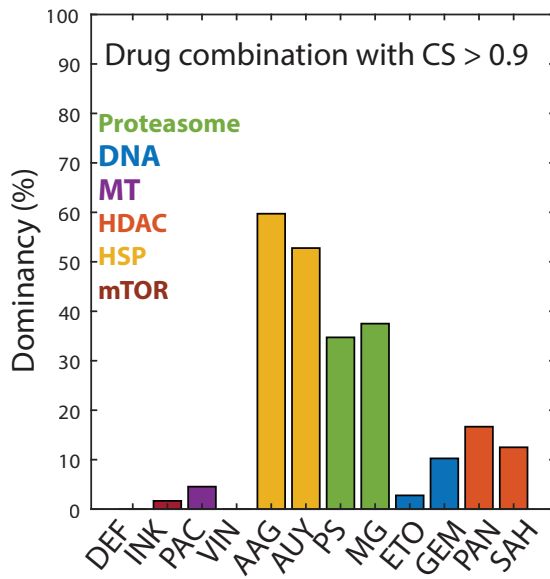

D

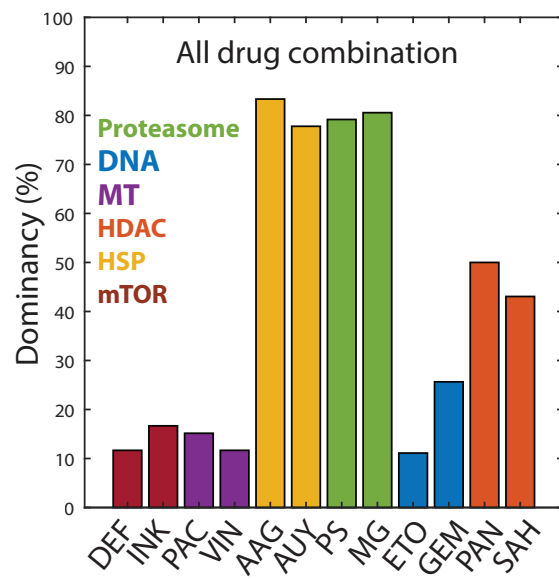

Supplement: Supplementary file 4 — Additional file 4: Sup Figure 4. (A) Venn diagram summary of drug synergy screen. Drug synergy was determined by three different methods, Loewe additivity, Bliss independence, and area under curve. Among 66 combinations, 8 combinations were synergistic by Loewe additivity, 6 by Bliss independence, and 41 by AUC. 3 combinations are synergistic by all three methods. (B) Histogram of confidence score. Confidence scores of KNN assignment for all combinations at 48-hr timepoint were shown in a histogram. KNN assignments with dominant behavior were shown in brown. (C-D) Drug dominancy statistics of 48-hr timepoint data set. Percentages of drug combinations showing dominant behavior was shown in a bar graph, using drug combinations with CS higher than 0.9 confidence score (C) or all combinations (D). 12 drug names: INK128 (INK), paclitaxel (PAC), vinblastine (VIN), 17AAG (AAG), AUY922 (AUY), PS341 (PS), MG132 (MG), etoposide (ETO), gemcitabine (GEM), panobinostat (PAN). [file 12885_2024_12057_MOESM4_ESM.pdf]

**A**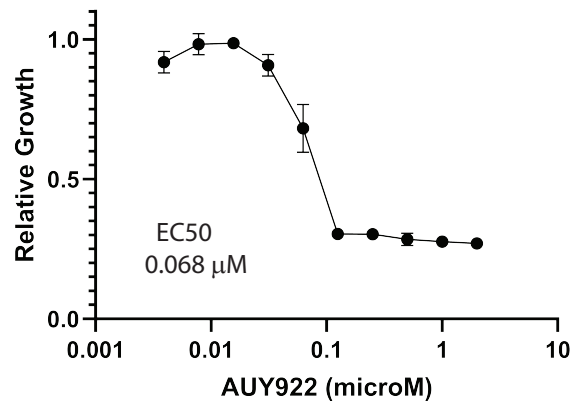**B**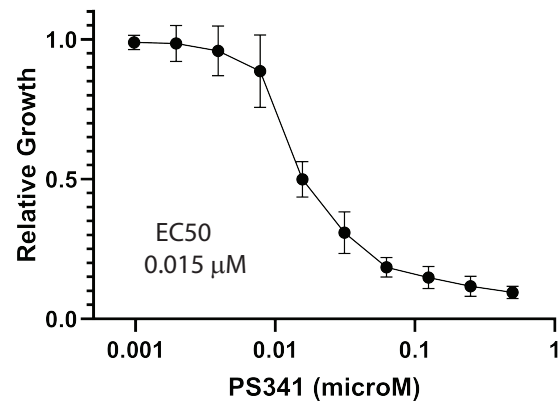**C**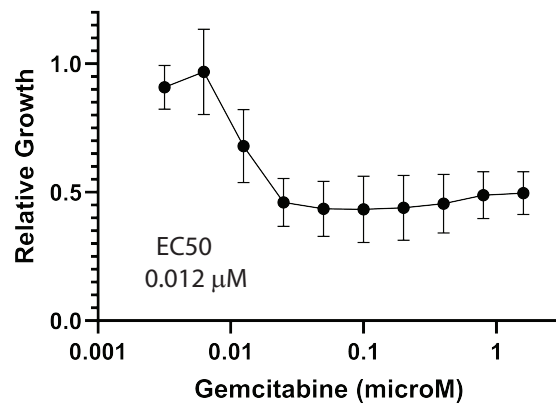**D**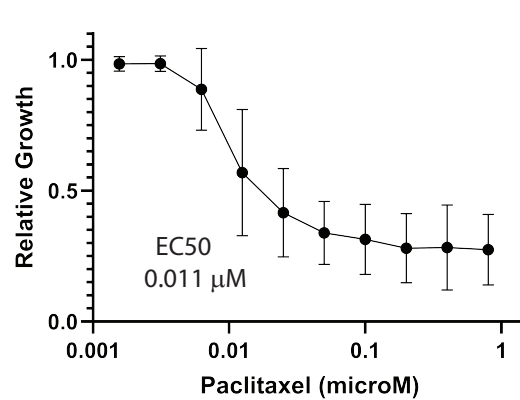**E**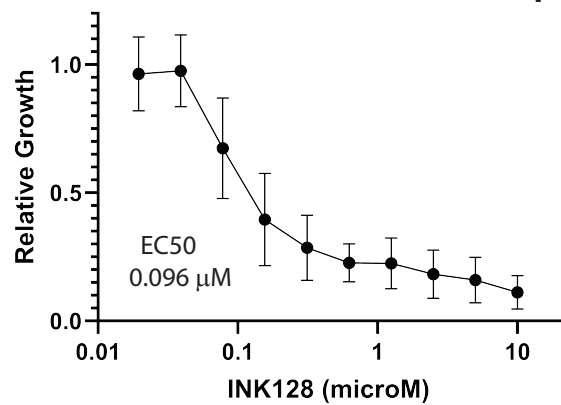**F**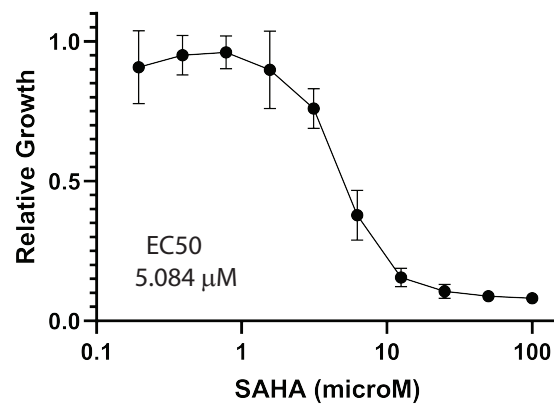**G**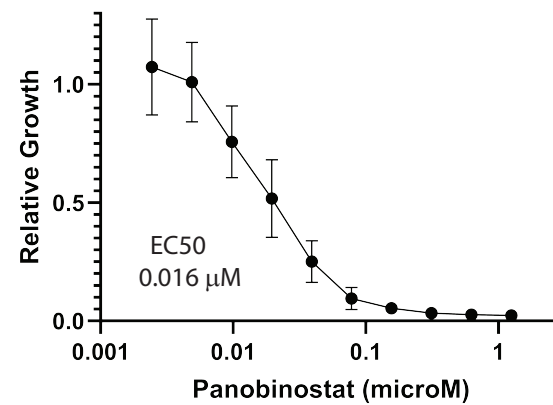

Supplement: Supplementary file 5 — Additional file 5 Sup Figure 5. Relative growth curves of single drug treatment. Cells were treated with (A) AUY922, (B) PS341, (C) gemcitabine, (D) paclitaxel, (E) INK128, (F) SAHA, or (G) panobinostat, for 3 days and cell viability was measured by CCK8 luminescent assay. Relative growth value was determined by comparing their growth with DMSO-treated cells or blank media. Mean values of relative growth were plotted with standard deviation (n=4). EC50 values were calculated by GraphPad prism. [file 12885_2024_12057_MOESM5_ESM.pdf]

EC20

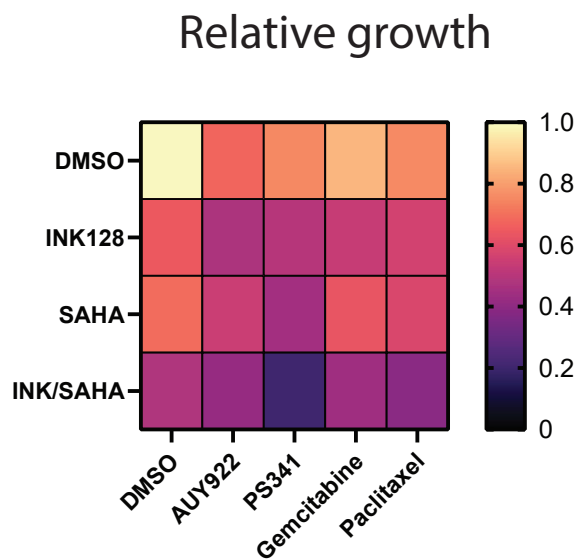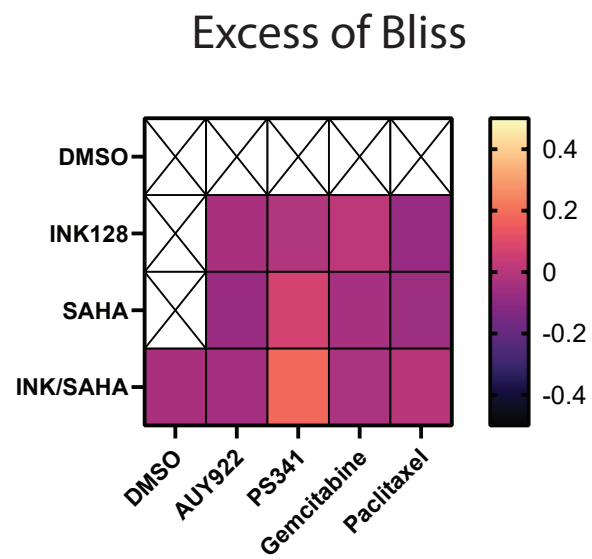

EC50

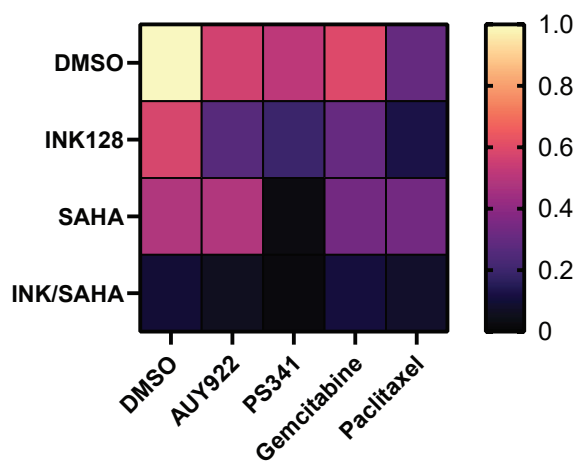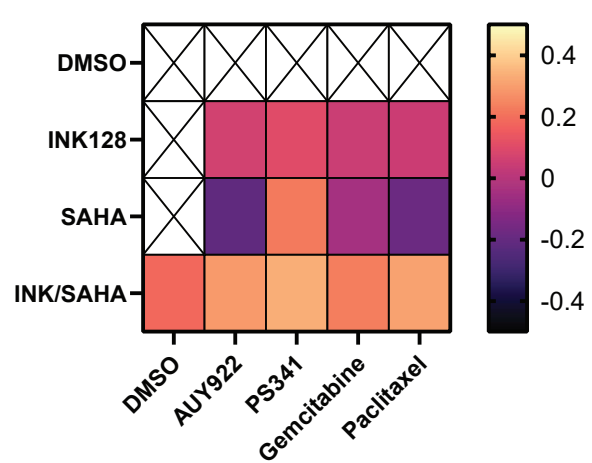

Supplement: Supplementary file 6 — Additional file 6: Sup Figure 6. Relative growths after drug combination treatmentd and their EOBs. EC20 or EC50 values of each single drug were first deduced from growth rate curve (Sup Figure 5) by GraphPad prism and adjusted values after verification were used. EC20 and EC50: INK128 (17 nM & 96 nM), SAHA (1.5 µM & 5 µM), AUY922 (31 nM & 68 nM), PS341 (10 nM & 15 nM), Gemcitabine (12 nM & 37 nM), and Paclitaxel (50 nM & 500 nM). Drug mixture (single, double or triple) using EC20 level of each drug was then treated to cells for 3 days and cell viability was measured by CCK8 luminescent assay. Relative growth value was determined by comparing their growth with DMSO-treated cells or blank media and EOB was accordingly calculated. Mean values of relative growth (n=4) and EOBs were shown as a heatmap. [file 12885_2024_12057_MOESM6_ESM.pdf]
